# A common *IGF1R* gene variant predicts later life breast cancer risk in women with preeclampsia

**DOI:** 10.1007/s10549-022-06789-9

**Published:** 2022-11-04

**Authors:** Mark Powell, Sophia Fuller, Erica Gunderson, Christopher Benz

**Affiliations:** 1 Buck Institute for Research On Aging, 8001 Redwood Blvd, Novato, CA 94945 USA; 2 Graduate Group in Biostatistics, School of Public Health, University of California, Berkeley, CA USA; 3 Division of Research, Kaiser Permanente Northern California, Oakland, CA USA

**Keywords:** Preeclampsia, Breast cancer, IGF1R, Hormone receptor-positive, SNP, rs2016347

## Abstract

**Purpose:**

Preeclampsia has been inconsistently associated with altered later life risk of cancer. This study utilizes the Nurses’ Health Study 2 (NHS2) to determine if the future risk of breast and non-breast cancers in women who experience preeclampsia is modified by carrying a protective variant of rs2016347, a functional insulin-like growth factor receptor-1 (*IGF1R)* single nucleotide polymorphism.

**Methods:**

This retrospective cohort study completed within the NHS2 evaluated participants enrolled in 1989 and followed them through 2015, with a study population of 86,751 after exclusions. Cox proportional hazards models both with and without the impact of rs2016347 genotype were used to assess the risk of invasive breast cancer, hormone receptor-positive (HR+) breast cancer, and non-breast cancers.

**Results:**

Women with preeclampsia had no change in risk of all breast, HR+ breast, or non-breast cancers when not considering genotype. However, women carrying at least one T allele of rs2016347 had a lower risk of HR+ breast cancer, HR 0.67, 95% CI: 0.47–0.97, *P* = 0.04, with interaction term *P* = 0.06. For non-breast cancers as a group, women carrying a T allele had an HR 0.76, 95% CI: 0.53–1.08, *P* = 0.12, with interaction term *P* = 0.26.

**Conclusions:**

This retrospective cohort study found that women with preeclampsia who carry a T allele of *IGF1R* rs2016347 had a reduced future risk of developing HR+ breast cancer, and a reduced but not statistically significant decreased risk of non-breast cancers suggesting a possible role for the IGF-1 axis in the development of cancer in these women.

**Supplementary Information:**

The online version contains supplementary material available at 10.1007/s10549-022-06789-9.

## Introduction

Preeclampsia develops in 5–12% of all pregnancies depending on race, ethnicity, and other risk factors within the population and is a leading cause of maternal/fetal perinatal mortality as well as being linked with later life maternal cardiovascular disease (CVD) [1]. Much less widely accepted, however, is that preeclampsia may protect against later life maternal breast cancer development, as the evidence is mixed depending on specific cohort characteristics and whether or not a woman’s inherited genotype for the common insulin-like growth factor receptor-1 (*IGF1R*) gene variant rs2016347 is also considered [2–4]. Further validation and mechanistic insight into the breast cancer protective impact of preeclampsia as influenced by this functional *IGF1R* single nucleotide polymorphism (SNP) might immediately improve breast cancer risk assessment and personalized screening, as well as suggest new breast cancer prevention strategies.

Preeclampsia is characterized by the development of high blood pressure after the 20th week of gestation plus the presence of proteinuria or other end organ dysfunction, which distinguishes it from gestational hypertension, the other major component of the broader class of disorders known as hypertensive disorders of pregnancy (HDP) [5, 6]. The association of preeclampsia with increased maternal risk of future CVD and mortality is now well-established [7, 8]. An association of preeclampsia with lower breast cancer risk was initially reported decades ago, and many subsequent large cohort studies have shown 10–20% lower relative risk, although these findings have not been universal [9–13]. The association of preeclampsia with later life risk for developing other cancers has also yielded inconsistent results overall and for specific cancer subtypes [14–18].

The systemwide sequelae of preeclampsia have long been noted and debated; and while preeclampsia is still primarily considered a placental disorder its exact pathogenesis remains unclear. Postulated systemic impacts include vascular ischemia, immune dysfunction, oxidative stress, and dysregulated angiogenesis, along with derangements of the insulin-like growth factor-1 (IGF-1) axis [5, 19]. With regard to the latter, several reports have described low IGF-1 blood levels as well as decreased placental expression of IGF-1 in women with preeclampsia, and this has been hypothesized to play a role in the future risk of breast and other cancers [20–25].

IGF-1 plays an essential role in normal breast development; yet upregulation of the IGF-1 axis and increased IGF1R expression appear to be associated with the development of breast cancer [26–29]. A pooled analysis of 17 studies demonstrated that higher levels of circulating IGF-1 are associated with increased risk of breast cancer, with this being especially true for hormone receptor-positive (HR+) breast cancer [30]. Similarly, elevated serum levels or increased expression of IGF-1 has been implicated in the development of various non-breast cancers [31–35]. Of mechanistic relevance to the present study, the normal breast tissue of women with a history of preeclampsia who also inherit the lower expressing T allele of *IGF1R* rs2016347 exhibits increased mammary gland involution, a histologic predictor of reduced breast cancer risk [36].

Prebil in 2014 first reported that women with HDP whose germlines possess the T allele of rs2016347 had significantly lower mammographic density, a clinically recognized proxy for breast cancer risk [37]. This was postulated to result from the fact that inheritance of the functional rs2016347 T allele had been shown to be associated with significantly lower IGF1R mRNA expression in various healthy organs including the breast [38]. A subsequent case–control analysis within the California Teachers Study (CTS) demonstrated a decreased risk of breast cancer of over 60% in women with preeclampsia carrying the rs2016347 T allele, with a larger protective effect seen against HR+ breast cancer development [2]. However, a more recent UK Biobank analysis of women with a history of HDP did not find a lower risk for developing breast cancer in those carrying the T allele, although this study did not include specific preeclampsia history or data on HR+ breast cancer [18]. Others have shown that the outcome impact of the rs2016347 T allele is greater in women diagnosed with HR+ breast cancers [39, 40]. When looking at non-breast cancers, the UK Biobank study did demonstrate that women with HDP carrying the protective T allele had a statistically significant 41% lower risk of developing non-breast cancers, while there was no impact on cancer risk by HDP or the T allele alone [18].

Given the above inconsistent population-based evidence, we turned to the large and well-studied longitudinal Nurses’ Health Study 2 (NHS2) with its specific data on preeclampsia, HR+ status, and non-breast cancer outcomes to confirm the potentially important modulating impact of rs2016347 T allele inheritance on a woman’s future risk of developing cancer.

## Methods

### Study population and design

**Fig. 1:**
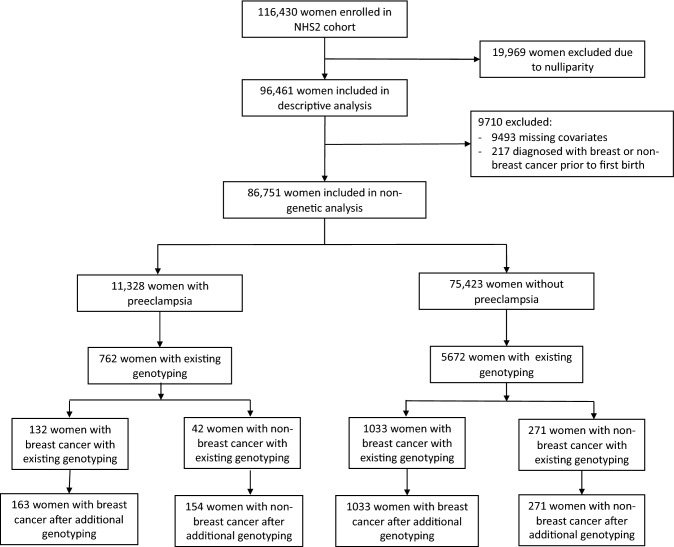
Study flow chart

This retrospective cohort study was completed within the NHS2, which enrolled 116,430 female registered nurses in 1989 with ages at entry ranging from 25 to 42. The original detailed entry questionnaire collected extensive data as did subsequent follow-up biennial questionnaires (available at https://nurseshealthstudy.org/participants/questionnaires) including preeclampsia history and necessary covariates such as age at entry, ethnicity, Body Mass Index (BMI), parity, age at first birth, age at menarche, family history of breast cancer, diet, physical activity, and smoking history. Covariates were modeled as continuous except for ethnicity, age at menarche, smoking history, and family history of breast cancer which were categorical with nominal categories as described in Table 1. Many papers detail the NHS2 and its extensive contributions to medical research [41–43]. The retrospective cohort sample for this study excluded 19,969 participants who were nulliparous, 9493 who had missing covariates, and 217 who developed a breast or a non-breast cancer prior to their diagnosis of preeclampsia leaving a final study number of 86,751. A subset of participants provided blood and buccal DNA samples for potential genotyping resulting in available information for our *IGF1R* SNP of interest, rs2016347, on 6577 participants; details on the genotyping process are provided below. A flow chart of study numbers is presented in Fig. 1. The NHS2 study protocol was approved by the Institutional Review Board (IRB) of the Brigham and Women's Hospital, and the IRB allowed participants’ completion of questionnaires as implied consent.

**Table 1:** Characteristics of NHS2 participants by preeclampsia status^a^

Characteristic	Preeclampsia + *N* = 11,328	Preeclampsia −*N* = 75,423
Age at entry	34.3 (4.6)	34.4 (4.7)
Race/ethnicity		
White non-Hispanic	10,550 (93.1%)	70,665 (93.7%)
Other	778 (6.9%)	4,758 (6.3%)
BMI at entry^**b**^	26.2 (5.9)	23.6 (4.5)
Parity	2.40 (1.05)	2.41 (1.04)
Age at first birth	26.0 (4.7)	26.6 (4.8)
Age at menarche		
< = 11	3,380 (29.8%)	17,348 (23.1%)
12–13	6,257 (55.2%)	43,965 (58.3%)
14+	1,653 (15.0%)	13,894 (18.4%)
Smoking history		
Never	7,319 (64.6%)	49,770 (66.0%)
Past/current	4,009 (35.4%)	25,653 (34.0%)
Family history of breast cancer	1,607 (14.2%)	10,949 (14.5%)
Diet (HEI)^**c**^	53.8 (10.3)	55.2 (10.5)
Physical activity (METS/week)^**d**^	20.5 (20.3)	22.3 (20.8)

^*a*^Mean (standard deviation); *n* (%)

^b^*BMI* body mass index

^c^*HEI* healthy eating index

^*d*^*METS/week* metabolic equivalents per week

### Study exposures and outcomes

Assessment of the primary exposure of preeclampsia was by self-report. Participants were asked if they had experienced preeclampsia in any pregnancy on their entry questionnaire in 1989 and on subsequent biennial questionnaires through 2001. Preeclampsia was defined as hypertension in pregnancy plus proteinuria on the entry questionnaire and as “preeclampsia/toxemia” on the follow-up biennial questionnaires, and participants responding yes on any questionnaire were deemed to have had a positive history of preeclampsia.

Outcome diagnoses of invasive breast cancer and non-breast cancers (defined as all cancers except breast and non-melanoma skin cancers) were determined by self-report with subsequent validation by NHS2 staff who obtained consent to review medical records. HR (±) breast cancer status was determined from medical records and/or pathology reports. Participants with HR− breast cancer or unclear HR status were excluded entirely from the HR+ breast cancer analyses, and the low number of documented HR− cases precluded their analysis as a subgroup.

### Samples for genotyping

In order to obtain genetic data for the NHS2, blood samples were collected from 29,611 and buccal smears from 29,392 participants from which DNA was extracted using Qiagen PureGene DNA Isolation Kits (Gentra Systems, Minneapolis, MN). Initial genotyping results of rs2016347 by the NHS2 was available on 6434 (7.4%) of study participants utilizing at least one of five different DNA analysis platforms as previously reported [44, 45]. The initial genotyping was performed entirely in 6 case control studies and preeclampsia disease status was not a selection criterion in any of these studies. The diseases studied and resulting genotyped participants included in the current study include breast cancer (*n* = 2,560), post-traumatic stress disorder (*n* = 1,553), endometriosis (*n* = 1,461), nephrolithiasis (*n* = 452), venous thromboembolism (*n* = 295), and ovarian cancer (*n* = 113). Of note, in the initial genotyping attempts were made to genotype all cases of breast cancer at that time.

In an effort to increase power for the genetic analyses, participants with preeclampsia who subsequently developed invasive breast cancer (*n* = 50) and had DNA available for genotyping but had not been initially genotyped had their DNA sent to the Brigham and Women’s Hospital/Harvard Cohorts Biorepository for rs2016347 genotyping. Similarly, 138 participants with preeclampsia and non-breast cancers with available DNA had genotyping requested. Because of limitations in DNA quantity/quality, only 31 of the 50 breast cancer specimens and 112 of the 138 non-breast cancer specimens were successfully genotyped, resulting in a final total of 6577 participants with rs2016347 genotyping. Among women with preeclampsia, these included 163 for all breast cancers, 126 for HR+ breast cancers, and 154 for non-breast cancers. The non-breast cancers consisted of 25 cases each of uterine cancer and melanoma, 24 cases of thyroid cancer, 15 of colon cancer, 14 with ovarian cancer, with all other cancer types contributing less than 10 cases each. Genotyping of rs2016347 among study participants was in Hardy Weinberg Equilibrium (*P* = 0.21), with an overall T/G allele frequency of 0.52/0.48.

### Genetic model selection

An a priori decision was made to focus on the dominant model in these analyses based on results of the impact of the rs2016347 genotype on previously studied outcomes, and with a desire to maximize statistical power while limiting potential multiple testing issues. The results of earlier published studies of the associations of rs2016347 with similar disease outcomes have demonstrated that the GT genotype tracks most closely with the TT genotype suggesting that only one T allele is necessary for risk modification [18, 37, 39, 40]. As our current analyses and hypothesis is focused on the potential “protective effect” of carrying a T allele, our results report the risk for those participants carrying at least one T allele (TT and GT combined) compared to the reference group of GG carriers.

### Statistical analysis

Hazard ratios (HR) and 95% confidence intervals (CI) for the association of preeclampsia with cancer outcomes were estimated from Cox proportional hazards models using person-years of time as the time scale. Person-years began accumulating for each of the years the participant remained in the study from the year of their first birth, and stopped accumulating upon the year of the final questionnaire (2015), year of their cancer diagnosis, loss to follow-up, or death, whichever occurred first. Participants that had a breast or non-breast cancer diagnosis after their first pregnancy but before study entry were included in the study as dates of cancer diagnosis were deemed adequately reliable to assure proper temporality. All participants with a breast or non-breast cancer diagnosis prior to preeclampsia history were excluded from the study. Breast cancer models are adjusted for age, ethnicity, BMI, parity, age at first birth, smoking history, family history of breast cancer, physical activity and diet (Healthy Eating Index); non-breast cancers for age, ethnicity, BMI, smoking, and diet. Data for alcohol intake was inadequate for utilization as a covariate. Proportional hazards assumptions for the models were evaluated using standard model-based methods [46]. Cox proportional hazard models were also used to estimate HRs and 95% confidence intervals for the association of genotype and cancer outcomes stratified by preeclampsia history. Linear interaction was then evaluated comparing the risk of each outcome for GG to GT/TT genotype of rs2016347 among those with preeclampsia history. The genetic models were adjusted for the same covariates used in the non-genetic models. Analyses were conducted using R version 4.2.0 “Vigorous Calisthenics”, and “survival” and “stats” packages were used to estimate hazard ratios and 95% confidence intervals [47–49]. Visualizations were created using “ggplot2”, and “gtummary” was used to create tables and figures [50, 51]. All code needed to reproduce the analyses is available on Github at https://github.com/sfuller2/tox-analysis.

## Results

### Participant characteristics

Characteristics of study participants by preeclampsia exposure status are presented in Table 1. Women with preeclampsia were slightly less likely to be White (White non-Hispanic), 93.1% vs 93.7%, had a lower age at menarche, and were slightly more likely to have smoked. They had a younger mean age at first birth of 26.0 vs 26.6 years, a higher BMI of 26.2 vs 23.6, a lower level of physical activity of 20.8 METS per week vs 22.7, and ate a less healthy diet than participants without preeclampsia. Both obesity and non-White race are well-established risk factors for preeclampsia [52].

Of participants in the analyses, 3133 (3.6%) had a history of breast cancer, 2149 (2.5%) had a history of HR+ breast cancer, and 3459 (4.0%) had a history of a non-breast cancer.

Comparisons of the characteristics of participants with all breast cancers, HR+ breast cancer only, and non-breast cancers compared to participants without are provided in Supplemental Tables S1–S3, respectively. Participants with all breast or HR+ breast cancer were older at entry, had lower parity, higher age at first birth, stronger family history of breast cancer, increased smoking history, decreased physical activity, and a slightly better diet than those without breast cancer. Participants with non-breast cancers were older, more likely to be White, have a smoking history, a higher BMI, and did less physical activity than participants without.

### Risks for breast and non-breast cancers in women with preeclampsia

**Fig. 2:**
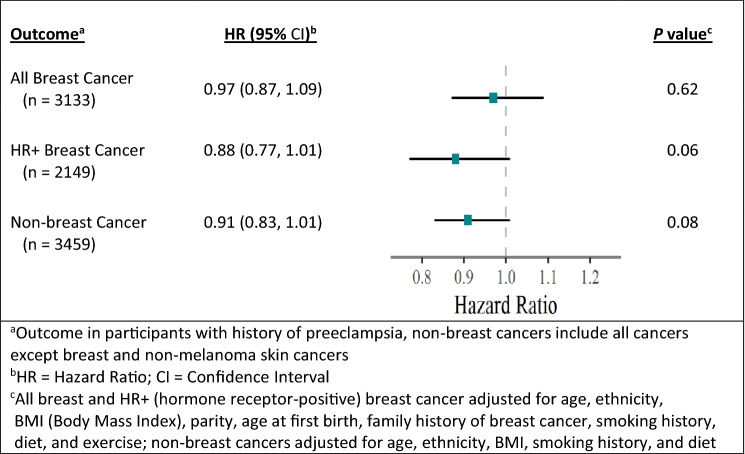
Outcome risk for NHS2 participants with preeclampsia

Women with a history of preeclampsia had no increase in risk of developing breast cancer when not considering genotype, HR 0.97; 95% CI, 0.87–1.09; *P* = 0.62. When looking at the subgroup of HR+ breast cancers, the risk decreased somewhat, HR 0.88; 95% CI, 0.77–1.01, *P* = 0.06. Assessment of the risk of developing a non-breast cancer revealed an HR 0.91; 95% CI, 0.83–1.01, *P* = 0.08. These results are summarized in Fig. 2.

### Association of IGF1R rs2016347 genotype with study outcomes

**Fig. 3:**
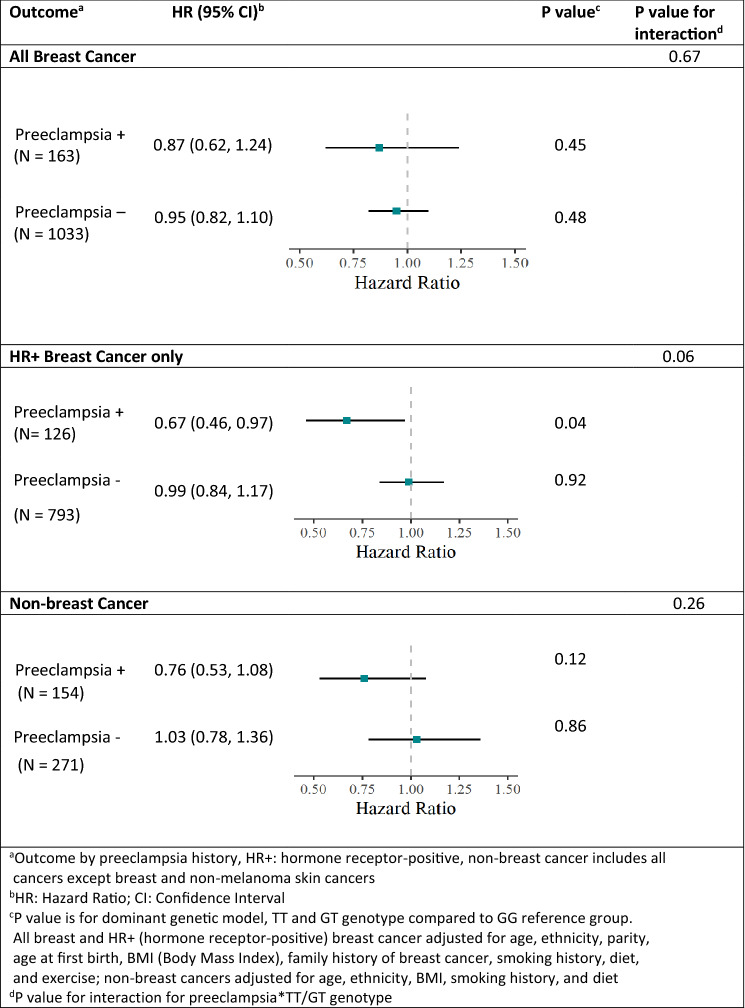
Outcome risk for NHS2 participants carrying at least one T allele of *IGF1R* SNP rs2016347 by preeclampsia history

The association of rs2016347 genotypes with the outcomes of breast, HR+ breast, and non-breast cancers did not reveal any significant association in participants without preeclampsia, as noted in Table 2. Similarly, risk for the dominant genetic model revealed no significant associations (see Fig. 3).

**Table 2:** Association of rs2016347 genotype with outcomes among participants without preeclampsia

*IGF1R* SNP rs2016347 genotype^**a**^				
Outcome^**b**^	GG	GT	TT	*P*-value^**c**^
Breast cancer (*N* = 1033)	24.5%	49.0%	26.5%	0.69
HR + Breast cancer (*N* = 793)	23.7%	49.3%	27.0%	0.92
Non-breast cancer (*N* = 271)	23.6%	46.9%	29.5%	0.78
No cancer (*N* = 4368)	23.5%	48.8%	27.7%	-----

^a^Values represent percent of each genotype with specified outcome

^b^N is the total number of participants with genotyping for each outcome

^c^P-values are for *χ*^2^ goodness-of-fit test using distribution of genotypes for no cancer as reference

group

### Impact of rs2016347 genotype on risk of cancer by preeclampsia status

There was no statistically significant change in risk for cancer in women with a history of preeclampsia when not considering *IGF1R* SNP rs20162347 genotype, although the impact on HR+ breast cancer was suggestive. Analyses stratified by preeclampsia history do demonstrate that genotype may in fact further influence risk. When looking at the risk for all breast cancers, carrying at least one T allele resulted in an HR 0.87; 95% CI, 0.62–1.24, *P* = 0.45. However, when looking at HR+ breast cancers only, carrying a T allele was protective, HR 0.67, 95% CI, 0.46–0.97, *P* = 0.04, with an interaction term for Preeclampsia*T allele of HR 0.68, 95% CI, 0.45–1.02, *P* = 0.06. Efforts to quantify the relative excess risk due to interaction (RERI) required removal of the additional targeted genotyping (31 of the 126 cases of HR+ breast cancer), and was suggestive of substantial interaction with an additive RERI of −0.46; 95% CI, −1.13–0.22, and multiplicative RERI of 0.69; 95% CI, 0.43–1.10. There were too few adequately documented HR− genotyped participants for meaningful analysis.

When looking at the risk of developing a non-breast cancer in women with preeclampsia, carrying at least one T allele resulted in an HR 0.76, 95% CI, 0.53–1.08, *P* = 0.12, with an interaction term for Preeclampsia*T allele HR 0.77, 95% CI, 0.49–1.21, *P* = 0.26. Results of the impact of rs2016347 for the dominant model are provided in Fig. 3.

Risks for all breast cancer, HR+ breast cancer only, and non-breast cancer demonstrated no impact from carrying a T allele in women without a history of preeclampsia, with HR 0.95, 95% CI, 0.82–1.10, *P* = 0.48, HR 0.99, 95% CI, 0.84–1.17, *P* = 0.92, and HR 1.03, 95% CI, 0.78–1.36, *P* = 0.86, respectively, as noted in Fig. 3. Of note, participants with genotyping did not differ in their risk of breast and non-breast cancer from the entire study population.

## Discussion

Results of this NHS2 study of predominantly White (> 90%) women demonstrate a 33% lower risk of developing HR+ breast cancer in those with a self-reported history of preeclampsia who also inherited the protective *IGF1R* T allele of rs2016347. Without considering inheritance of this functional *IGF1R* SNP, these women exposed to preeclampsia, much like those in the CTS initially evaluated without *IGF1R* genotyping, would otherwise appear to show no significant reduction in their later life breast cancer incidence (Fig. 2). Likewise, reviews of multi-national cohort studies performed before awareness of this *IGF1R* SNP contribution concluded that the general breast cancer risk-reducing impact of preeclampsia is modest (10–20%), if present at all [10]. Even today, for understudied populations like Black women whose age-adjusted prevalence of preeclampsia can exceed 12%, the question of preeclampsia’s potential protective effect on later life breast cancer incidence remains incomplete, as these studies have not accounted for *IGF1R* rs2016347 genotype differences [1].

Our current NHS2 findings, as well as those from the prior CTS study, now strongly suggest that the degree of breast cancer protection from preeclampsia depends on a mechanistic interaction between two population exposures: preeclampsia and inheritance of the functionally blunted *IGF1R* T allele of rs2016347 that causes a significant reduction in IGF1R mRNA expression observable in multiple normal organ and host tissues [38]. Moreover, both NHS2 and CTS studies also demonstrate that this protective mechanistic interaction appears strongest against HR+ breast cancer, producing a 30–40% reduction in this most common subtype of breast cancer. Furthermore, these concordant NHS2 and CTS findings are consistent with numerous preclinical and clinical studies examining the role of the IGF-1 axis in driving HR+ breast cancers [27–30]. At a mechanistic level, it is now well-established that tumorigenic crosstalk occurs between the estrogen receptor (ER) axis and epithelial signal transduction downstream of activated IGF1R, and that this crosstalk plays a role in both HR+ breast cancer development and progression [52–55]. Our findings are also consistent with those of Winder who found better survival outcomes for women with HR+ breast cancers treated with the anti-estrogen tamoxifen who carry the T allele of rs2016347 [40], as well as with those of Bhargava who found that while IGF1R overexpression is most common in HR+ relative to HR− breast tumors, patients with HR+ breast cancers expressing lower levels of IGF1R have significantly better survival outcomes following ER-targeted endocrine therapy [56].

Many population studies are unable to discern HR+ from HR− breast cancer outcomes, and this likely explains why a recent UK Biobank cohort analysis which lacked data on HR status was unable to show a significant lowering of overall breast cancer risk in women with HDP carrying the rs2016347 T allele [38]. However, that same UK Biobank study did detect a statistically significant 41% reduction in the development of non-breast cancers as a pooled group with > 40% comprising either colorectal, uterine or ovarian cancers; this observation strongly suggests that the cancer protecting mechanistic interaction between preeclampsia and T allele inheritance may extend to endocrine-independent tumor types beyond HR+ breast cancer. Our current NHS2 study suggests a 24% reduction in development of another pooled group of non-breast cancers (Fig. 3), in which 35% consisted of colorectal, uterine, or ovarian cancers. In summary, beyond our conclusive findings about protection against HR+ breast cancers, our observations about non-breast cancer outcomes suggest that future studies be performed to quantitate the non-breast cancer protective interactions between preeclampsia and *IGF1R* rs2016347 T allele inheritance in populations statistically powered to assess ovarian, colorectal and uterine cancer development.

A limitation of this study may have been that preeclampsia exposure was determined by self-report, yet studies of maternal recall of preeclampsia have found that misclassification is minimal; and a validation study of preeclampsia in the NHS2 demonstrated a positive predictive value of 89% [57–59]. As well, any misclassification resulting from recall bias would more likely be non-discriminatory due to the lack of awareness of any causal association between preeclampsia and cancer, and thus would be inclined to bias the results toward the null. Another limitation was that data linking preeclampsia to a specific pregnancy was sometimes lacking, and as a result, date of first pregnancy was used in these cases as preeclampsia is known to be much more common in the first pregnancy [60]. Low numbers did not permit analysis of HR− breast cancers as a group, and data also did not allow analysis by subtypes of preeclampsia such as early/late, mild/severe, or preeclampsia superimposed on chronic hypertension. Genotyping for this study was not random, and although there was no selection based on preeclampsia and all breast cancer cases were genotyped, this could have introduced ascertainment bias which may have impacted the results. However, the genotype has not previously been associated with the outcome of any of the case control studies.

When comparing genotype-preeclampsia interactions on breast cancer risk between NHS2, UK Biobank and CTS cohort studies, it is important to note that rs2016347 allele frequencies are very similar in each of the 3 cohorts. However, differences in their population risk factors may play a role in their varied outcome risks. All three cohorts are largely White and match in most characteristics except age, with participants in the NHS2 being 20 years younger at study entry and thus potentially more likely to have developed premenopausal breast cancer [3, 18]. Likewise, this age difference accounts for differences in the number and type of tumors comprising our non-breast cancer group. In particular, the younger age of the NHS2 cohort resulted in a larger percentage of thyroid cancer and melanoma cases, and a lower percentage of colorectal cancer cases. In addition, the targeted genotyping of a small number of participants with ovarian cancer may also have contributed to the different HRs noted between the non-breast cancer groups for NHS2 (HR 0.76) and UK Biobank (HR 0.59) cohorts.

## Conclusion

This retrospective NHS2 cohort study in largely White non-Hispanic women validates earlier CTS findings and confirms that women with preeclampsia who have inherited the common T allele of *IGF1R* rs2016347 have a 30–40% lower risk of developing HR+ breast cancer, pointing to the need to consider incorporating this exposure information (preeclampsia history and *IGF1R* rs2016347 genotyping) into risk assessment models and personalized breast cancer screening recommendations. Importantly, this breast cancer protective interaction has yet to be validated in non-White women who have the highest exposure rates to preeclampsia and may be more likely to develop premenopausal HR− breast cancer. Of interest, this NHS2 study also observed a nominal reduction in non-breast cancer incidence, supporting the statistically significant UK Biobank cohort findings of reduced non-breast cancer development in women with both exposures. Taken together, these cohort studies strongly implicate a mechanistic role of the IGF-1 axis in cancer development, supporting targeted prevention strategies designed to blunt IGF-1 axis expression.

## Supplementary Information

Below is the link to the electronic supplementary material.

Supplementary file1 (DOCX 21 KB)

## Data Availability

The datasets utilized for this study and the code used for the analyses are available from the corresponding author upon reasonable request.
