# Supplementary material for: A common IGF1R gene variant predicts later life breast cancer risk in women with preeclampsia
Source: Breast Cancer Res Treat. 2022 Nov 4;197(1):149–59. doi: 10.1007/s10549-022-06789-9 (PMC9823040; doi:10.1007/s10549-022-06789-9)
Supplement: Supplementary file 1 — Supplementary file1 (DOCX 21 KB) [file 10549_2022_6789_MOESM1_ESM.docx]

| **Supplemental Table 1. Characteristics of Nurses’ Health Study 2 participants by breast cancer status^a^** | | | |
| --- | --- | --- | --- |
| **Characteristic** | **Breast Cancer +**  N = 3,133 | **Breast Cancer -**  N = 83,618 |  |
| **Preeclampsia** | 378 (12.1%) | 10,950 (13.1%) |  |
| **Age at entry** | 35.9 (4.4) | 34.4 (4.7) |  |
| **Race/ethnicity** |  |  |  |
| White non-Hispanic | 2,929 (93.5%) | 78,286 (93.6%) |  |
| Other | 204 (6.5%) | 5,332 (6.4%) |  |
| **BMI^b^ at entry** | 23.8 (4.6) | 24.0 (4.8) |  |
| **Parity** | 2.34 (0.96) | 2.41 (1.05) |  |
| **Age at first birth** | 26.9 (4.9) | 26.5 (4.8) |  |
| **Age at menarche** |  |  |  |
| <=11 | 812 (25.9%) | 19,916 (23.8%) |  |
| 12-13 | 1,806 (57.6%) | 48,416 (57.9%) |  |
| 14+ | 501 (16.0%) | 15,046 (18.0%) |  |
| **Smoking history** |  |  |  |
| Never | 1,961 (62.6%) | 55,128 (65.9%) |  |
| Past/current | 1,172 (37.4%) | 28,490 (34.1%) |  |
| **Family history of**  **breast cancer**  **Physical activity**  **(METS/week)**^c^  **Diet (HEI)**^d^ | 746 (23.8%)  20.5 (18.3)  55.7 (10.5) | 11,810 (14.1%)  22.1 (20.9)  55.0 (10.5) |  |
| ^a^Mean (Standard deviation); n (%) ^b^BMI: Body Mass Index | | | |

^c^METS: Metabolic equivalents per week

^d^HEI: Healthy Eating Index

| **Supplemental Table 2. Characteristics of Nurses’ Health Study 2 participants by HR+ breast cancer status**^a^ | | | |
| --- | --- | --- | --- |
| **Characteristic** | **HR+ Breast Cancer**  N = 2,149 | **No HR+ Breast Cancer**  N = 84,602 |  |
| **Preeclampsia** | 235 (10.9%) | 11,093 (13.1%) |  |
| **Age at entry** | 35.9 (4.4) | 34.4 (4.7) |  |
| **Race/ethnicity** |  |  |  |
| White non-Hispanic | 2,017 (93.9%) | 79,198 (93.6%) |  |
| Other | 132 (6.1%) | 5,404 (6.4%) |  |
| **BMI**^b^ **at entry** | 23.7 (4.5) | 24.0 (4.8) |  |
| **Parity** | 2.34 (0.95) | 2.41 (1.05) |  |
| **Age at first birth** | 26.9 (4.8) | 26.5 (4.8) |  |
| **Age at menarche** |  |  |  |
| <=11 | 545 (25.4%) | 20,183 (23.9%) |  |
| 12-13 | 1,240 (57.7%) | 48,982 (57.9%) |  |
| 14+ | 356 (16.6%) | 15,191 (18.0%) |  |
| **Smoking history** |  |  |  |
| Never | 1,335 (62.1%) | 55,754 (65.9%) |  |
| Past/current | 814 (37.9%) | 28,848 (34.1%) |  |
| **Family history of**  **breast cancer**  **Physical activity**  **(METS/week)**^c^  **Diet (HEI)**^d^ | 542 (25.2%)  20.4 (18.1)    56.1 (10.4) | 12,014 (14.2%)  22.1 (20.8)    55.0 (10.5) |  |
| ^a^HR+: Hormone receptor positive; Mean (Standard deviation); n (%) ^b^BMI: Body Mass Index | | | |

^c^METS: Metabolic equivalents per week

^d^HEI: Healthy Eating Index

| **Supplemental Table 3. Characteristics of Nurses’ Health Study 2 participants by non-breast cancer status**^a^ | | | |
| --- | --- | --- | --- |
| **Characteristic** | **Non-breast Cancer +**  N = 3,459 | **Non-breast Cancer -**  N = 80,546 |  |
| **Preeclampsia** | 449 (13.0%) | 10,565 (13.1%) |  |
| **Age at entry** | 35.8 (4.5) | 34.3 (4.7) |  |
| **Race/ethnicity** |  |  |  |
| White non-Hispanic | 3,309 (95.7%) | 75,332 (93.5%) |  |
| Other | 150 (4.3%) | 5,214 (6.5%) |  |
| **BMI**^b^ **at entry** | 24.9 (5.5) | 23.9 (4.7) |  |
| **Smoking history** |  |  |  |
| Never | 2,143 (62.0%) | 53,226 (66.1%) |  |
| Past/current  **Physical activity**  **(METS/week)**^c^  **Diet (HEI)**^d^ | 1,316 (38.0%)  20.6 (18.8)  54.8 (10.5) | 27,320 (33.9%)  22.2 (20.9)  55.0 (10.5) |  |
| ^a^Mean (Standard deviation); n (%) ^b^BMI: Body Mass Index | | | |

^c^METS: Metabolic equivalents per week

^d^HEI: Healthy Eating Index
